# Why do men extend their employment beyond pensionable age more often than women? a cohort study

**DOI:** 10.1007/s10433-021-00663-1

**Published:** 2021-12-05

**Authors:** Saana Myllyntausta, Marianna Virtanen, Jaana Pentti, Mika Kivimäki, Jussi Vahtera, Sari Stenholm

**Affiliations:** 1 Department of Psychology and Speech-Language Pathology, University of Turku, Turku, Finland; 2 Department of Public Health, University of Turku and Turku University Hospital, Turku, Finland; 3 Centre for Population Health Research, University of Turku and Turku University Hospital, Turku, Finland; 4 School of Educational Sciences and Psychology, Psychology, University of Eastern Finland, Joensuu, Finland; 5 Division of Insurance Medicine, Karolinska Institutet, Stockholm, Sweden; 6 Clinicum, Faculty of Medicine, University of Helsinki, Helsinki, Finland; 7 Department of Epidemiology and Public Health, University College London Medical School, London, UK; 8 Finnish Institute of Occupational Health, Helsinki, Finland

**Keywords:** Aging, Mediation analysis, Postponing retirement, Sex differences, Work characteristics

## Abstract

Men extend their employment beyond pensionable age more often than women, but the factors that contribute to this sex difference are unknown. This study aimed to examine sex differences in extending employment and the contribution of sociodemographic, work- and health-related factors to these differences. Participants of this prospective cohort study were 4,263 public sector employees from Finland who reached their individual pensionable date between 2014 and 2019 and responded to a survey on work- and non-work-related issues before that date. Extended employment was defined as continuing working for over six months beyond the individual pensionable date. We used mediation analysis to examine the contribution of explanatory factors to the association between sex and extended employment. Of the participants, 29% extended employment beyond the pensionable date. Men had a 1.29-fold (95% confidence interval 1.11–1.49) higher probability of extending employment compared with women. Men had a higher prevalence of factors that increase the likelihood of extended employment than women (such as spouse working full-time, no part-time retirement, low job strain, high work time control, and lack of pain) and this mediated the association of sex with extended employment by up to 83%. In conclusion, men were more likely to extend their employment beyond pensionable age than women. This difference was largely explained by men being more likely to have a full-time working spouse, low job strain, high work time control, no pain, and not being on part-time retirement.

**Supplementary Information:**

The online version contains supplementary material available at 10.1007/s10433-021-00663-1.

## Introduction

By 2050, the old-age dependency ratio is projected to be 52% in the European Union, indicating that there will be less than two persons of working age for each individual aged 65 years or over (Eurostat 2020). To tackle the reducing number of working-age people, many European countries aim to increase labor market participation (Rechel et al. 2013), including policies to extend working life by raising retirement age or encouraging employees to work beyond their pensionable age (Hess 2017; Ogg and Rasticová 2020). To motivate older employees to continue longer in working life, it is important to understand what factors are associated with extended employment.

In studies of public sector employees in Finland (Riekhoff and Järnefelt 2017; Virtanen et al. 2014, 2017) and the general working population in other European countries (Andersen et al. 2020; Wahrendorf et al. 2017), men have been observed to be more likely to work at older ages and to extend their employment beyond the pensionable age than women. However, it is not clear what factors contribute to this sex difference. One explanation could be the unbalanced distribution of family roles or responsibilities, with men being less likely than women to take over care responsibilities (Carr et al. 2018; Stoiko and Strough 2019). In agreement with this, men’s retirement timing has been found to more closely resemble women’s, when men enact caregiving roles (Stoiko and Strough 2019). On the other hand, women have typically lower levels of pension accrual than men as a result of working in lower-paid jobs and having lower average income (König 2017; Rantala and Riihelä 2016; Riekhoff and Järnefelt 2018), which might require some women to work longer to secure an adequate retirement income.

In addition to male sex, previous studies have identified other characteristics that predict working beyond pensionable age, including sociodemographic and health factors, as well as factors related to working conditions. Those with no spouse have been observed to be more likely to extend their employment beyond pensionable age or to work at an older age (beyond 65 years) (Nolan and Barrett 2019; Virtanen et al. 2014). Healthier people, in terms of both physical and mental health, are also more likely to work beyond pensionable age (de Wind et al. 2016, 2018; Demou et al. 2017; Scharn et al. 2017; Virtanen et al. 2014). Those in higher occupational classes have been shown to extend employment more often than those in lower occupational classes (Virtanen et al. 2017; Wahrendorf et al. 2017). Especially having lower physical workload, higher work time control, and better-perceived workability have been suggested to play role in the higher likelihood for prolonging employment among those in more advantaged occupational positions. In addition, good psychosocial working conditions such as high control overwork and low effort-reward imbalance have been shown to predict working beyond pensionable age (Browne et al. 2019; Wahrendorf et al. 2017), even among those with physically demanding jobs (Andersen et al. 2020). However, it is unclear whether all these factors are associated with extended employment similarly among men and women or whether differences in these factors contribute to sex differences in the likelihood of working beyond pensionable age. A further limitation in previous studies is a focus on early retirement or work participation at older ages (usually beyond age 65) (König 2017; Stoiko and Strough 2019; Weber et al. 2019), while prospective research on sex differences in regard to actual extension of employment beyond the pensionable age is scarce (Wahrendorf et al. 2017).

In this prospective cohort study of Finnish public sector employees, we aimed to quantify sex differences in extended employment beyond pensionable age. In addition, we sought to identify the contribution of sociodemographic, work- and health-related factors to these differences. Two different methods were compared: traditional mediation analysis (“the difference method”) (Baron and Kenny 1986) and counterfactual mediation analysis (Valeri and VanderWeele 2013).

## Methods

### Study population

Data from the Finnish Retirement and Aging study (FIREA) were used. FIREA is an ongoing longitudinal cohort study of aging public sector employees in Finland established in 2013. Detailed description of the study design has been provided elsewhere (Leskinen et al. 2018). The eligible population for the FIREA study cohort included all public sector employees whose individual pensionable date was between 2014 and 2019 and who were working in one of the 27 municipalities in Southwest Finland or the nine selected cities or five hospital districts around Finland in 2012 (n = 10,629).

Information on the participants’ official individual pensionable dates was obtained from the pension insurance institute for the public sector (Keva Public Sector Pensions). The participants reported their actual retirement date in questionnaires. Participants were first contacted by sending them a questionnaire 18 months prior to their individual pensionable date (i.e. the lowest age of eligibility for old-age pension). The questionnaire was thereafter sent to the participants annually, at least four times in total. By the end of December 2019, 6,783 cohort members (64% of the eligible sample) had responded to at least one questionnaire. In this study, we included participants who responded to the questionnaire at least once before the pensionable date between 2013 and 2019 and who either reported their actual retirement date or continued working beyond their individual pensionable date (for a minimum of six months) (n = 4,263). The FIREA study was conducted in line with the Declaration of Helsinki and it was approved by the Ethics Committee of Hospital District of Southwest Finland (84/1801/2014). All FIREA participants have given written informed consent to participate in the study.

### Assessment of extended employment

In Finland, the retirement ages in the public sector are regulated by the Public Sector Pensions Act. Until the end of 2016, each employee had an individual pensionable date. In general, public sector employees could retire at age 63, but they also could work beyond this age until they reached the age of 68 years, and working beyond the pensionable date accrued a higher pension. After a pension reform in January 2017, each age group has its own retirement age range and the lowest age of eligibility for old-age pension will gradually increase; the increase being 3 months/year until year 2030 after which the increase will be tied to the average life expectancy (Keva 2018). Thus, the retirement age is flexible within a certain age range, being for example from 63 years and 3 months until turning 68 years for those born in 1955 and from 65 years until turning 70 years for those born in 1962. In addition, for some employees, such as primary school teachers, it was possible to keep their earlier retirement age from the previous pension acts in which it was below 63 years. When an employee reached the statutory retirement age window and decided to retire, his/her pension depended on the age at retirement, as the amount of the pension depended on the duration of the working career and the salary with working longer accumulating a higher pension. Information on participant’s pension scheme (i.e. whether the individual pensionable date was before or after the pension reform in 2017) was treated as a covariate in the analyses.

First, the time between the register-based individual pensionable date and the actual retirement date was first calculated. Participants were then divided into two groups: (1) those who did not extend their employment or extended it six months at the most beyond the individual pensionable date (i.e. no extension) and (2) those who extended their employment for over six months beyond the pensionable date (i.e. extended employment). This cut-off point for the extension of employment was chosen as the same cut-off point has been used in previous studies examining extended employment among Finnish employees (Virtanen et al. 2014, 2017). If the participant had not retired at the time of the last available survey, but the follow-up time between the pensionable date and the date of completing the last survey was over six months, they were categorized into the extended employment group.

### Assessment of sex and potential explanatory factors

Information on sex and occupational titles of the last known occupation prior to pensionable date was obtained from Keva Public Sector Pensions. We use the term “sex” throughout the text, as the information was register-based, although psychosocial and cultural factors are also considered. Occupational titles were coded based on the Standard Classification of Occupations (ISCO) by Statistics Finland (2019) and occupational status was categorized into two groups: non-manual occupations (ISCO classes 1–4, e.g. teacher, physicians, registered nurses) and service and manual occupations (ISCO classes 5–9, e.g. cleaners, maintenance workers). A validated gender-specific job exposure matric was used to estimate physical workload at each occupation (low physical workload vs. no) (Solovieva et al. 2012).

Information on the other potential explanatory factors was derived from the last FIREA questionnaire preceding the individual pensionable date. We examined the following sociodemographic and work factors: marital status (married vs. no; the latter including single, divorced/separated, and widowed), working status of a spouse (spouse working full-time vs. no; the latter including also those with no spouse), caregiving status (not providing care vs. providing care for a family member), part-time retirement (yes vs. no; based on self-reported employment status), shift work status (regular working hours vs. shift work; the latter including shift work with or without night shifts, regular night work, and other irregular work), and good working capacity (yes vs. no; measured on a scale from 0 [i.e. incapable of working] to 10 [i.e. the best possible working capacity] with scores 8–10 presenting good working capacity (Ilmarinen et al. 1997)).

Information on job strain and work time control was also included. Job strain was measured using nine items assessing job control and five items assessing job demands from the short version of the Job Content Questionnaire (Fransson et al. 2012; Karasek et al. 1998). Job strain was defined as the difference between means scores of the job demand items and job control items (both evaluated on a 5-point Likert-type scale ranging from 1 = totally agree to 5 = totally disagree) and dichotomized into a measure of low job strain (yes, lowest tertile of the scores; no, the other two upper tertiles). Work time control was assessed by asking participants to evaluate how much they could influence several aspects of their working time (evaluated on a scale from 1 = very little to 5 = very much), such as the length of a workday, the starting and ending times of a workday, the handling of private matters during the workday, the scheduling of vacations and paid days off, and the taking of unpaid leave (Ala-Mursula et al. 2005). The highest tertile of the scores was set to indicate high work time control and the other two tertiles as not (yes vs. no). As the questions related to job strain and work time control were not included in FIREA study until the 2016 survey, we used additional information from another cohort study, the Finnish Public Sector (FPS) study (Kivimäki et al. 2007), in which most of the participants had also participated, for those with missing information on these factors who gave their permission to link their information from the FPS surveys (35% of the respondents).

In addition, the following factors associated with health and lifestyle were examined: self-rated health (good vs. suboptimal; assessed with a 5-point scale from 1 = good to 5 = poor with options 1 and 2 indicating good health), psychological distress (yes vs. no; based on a 12-item version of the General Health Questionnaire with a cut-off point of four or more symptoms indicating psychological distress (Goldberg 1972)), pain (no pain vs. mild or severe pain), chronic diseases (no chronic diseases vs. 1 or more chronic diseases, including coronary artery disease, myocardial infarction, stroke, intermitted claudication, hypertension, diabetes, osteoarthritis, osteoporosis, sciatica, fibromyalgia, rheumatoid arthritis, depression, other mental disorder, asthma, and cancer), self-reported sleep duration (≥ 6.5 h vs. less, based on usual sleep duration per 24 h), sleep difficulties (yes vs. no, based on the Jenkins Sleep Problem Scale (Jenkins et al. 1988) with a cut-off point of at least 4 nights/week indicating sleep difficulties), smoking status (non-smoker vs. currently smoking), alcohol use (no risk-use of alcohol vs. risk-use of alcohol; based on weekly alcohol use with the limit for risk-use set as > 288 g/week for men and > 192 g/week for women), physical activity (recommended physical activity vs. no, measured as the metabolic equivalent task [MET] hours with the cutoff point for recommended physical activity set as > 14 MET hours/week), and body mass index (normal weight vs. no; based on self-reported weight and height with the limit for normal weight set as < 25 kg/m^2^).

### Statistical analyses

Differences between men and women in sociodemographic, work- and health-related factors at baseline were examined using χ2 test. In addition, we examined the associations between these potential explanatory factors and working beyond the pensionable date separately for men and women using log-binomial regression models. Log-binomial regression analysis is recommended when the outcome is common, as in this case (29% with extended employment) (Valeri and VanderWeele 2013). The results are expressed as unadjusted risk ratios (RR) and their 95% confidence intervals (CI).

To examine the contribution of explanatory factors on the association between sex and extended employment we used mediation analysis. Since mediation analysis requires that the exposure (here sex) and the explanatory factor (i.e. the mediator) are correlated and that both are associated with the outcome (extended employment), we included only those baseline factors in the mediation analyses that were associated with sex in the total sample and extended employment in men, women, or both. To be included in the mediation analyses, all participants were required to have information on each of the factors that were used in the mediation analyses. This resulted in an analytic sample of 2,513 participants. In the mediation analysis, we first examined the association between sex and extended employment and then serially adjusted for each of the explanatory factors. We report the results as RRs and their 95% CIs. The models have been adjusted for pension scheme (i.e. whether participant’s individual pensionable date was before or after the pension reform in 2017). We calculated the percentage of excess risk mediated (PERM) by each factor as follows: PERM = [RR(pension scheme adjusted)—RR(explanatory factor adjusted)]/[RR(pension scheme adjusted)—1] × 100.

However, the traditional mediation analysis described above, based on the method by Baron and Kenny (1986), does not take into account that the exposure and mediator may interact. This possibility has been addressed by the new causal interference methods that are based on the counterfactual framework. Using the SAS macro presented by Valeri and VanderWeele (2013), we used the counterfactual mediation analysis that allows the presence of exposure-mediator interaction and decomposes the effects into direct and indirect effects. Natural direct effect (NDE) provides RR for the association between sex and extended employment in a hypothetical scenario where the level of exposure to a potential explanatory factor (the mediator) is similar among both men and women. Natural indirect effect (NIE) refers to the excess risk of extended employment among men compared to women that is due to their exposure to potential explanatory factors. In total effect (TE), both natural direct and indirect effects are considered to estimate the RRs for the association between sex and extended employment. The SAS macro produces also the proportion (%) of the TE that the mediator in question explains. We performed the counterfactual mediation analyses using dichotomous exposure, mediator, and outcome variables. The outcome was modeled using a log-binomial regression and the mediators by using logistic regression. All statistical analyses were performed with SAS 9.4 Statistical Package (SAS Institute, Cary, North Carolina, USA).

Finally, we conducted an additional analysis to assess the robustness of our findings by using a cut-off point of working over 12 months beyond the pensionable date. The association between the potential explanatory factors and the extension of employment of over 12 months was examined separately for men and women and those factors that were associated with both sex and this longer extension of employment were included in the mediation analyses. The analytic sample for the extension of employment of over 12 months was 2,819 participants with no missing data on the factors that were used in the mediation analyses.

## Results

The mean individual pensionable age was 63.8 years (SD 1.1) among women and 63.9 years (SD 1.3) among men. Of the participants, 2,177 (51%) had their individual pensionable date before the 2017 pension reform. We observed differences between men and women in many of the sociodemographic, work- and health-related factors at baseline, as seen in Table 1. In total, 1,233 (29%) participants extended their employment by more than six months beyond the pensionable date. Men were more likely to extend their employment (35%) than women (28%). The final study population (n = 4,236) was reasonably representative of the eligible population (n = 10,629) in terms of sex distribution (18% men vs. 20% in the eligible population), occupations (65% in non-manual occupation vs. 58%), and the mean individual pensionable age (63.8 years [SD = 1.2] vs. 64.1 years [SD 1.1]).

**Table 1:** Association of sociodemographic factors, work characteristics, and health-related factors before retirement with sex

Characteristics	All (n = 4263)	Men (n = 762)	Women (n = 3501)	*p* for difference
	n (%)	n (%)	n (%)	
Married or cohabiting	2925 (71)	616 (83)	2,309 (68)	< .0001
Spouse working full-time	902 (22)	320 (43)	582 (17)	< .0001
Not providing care for a family member	3528 (85)	648 (87)	2880 (84)	0.055
Non-manual occupation	2739 (65)	518 (68)	2221 (64)	0.022
Part-time retirement	866 (20)	123 (16)	743 (21)	0.002
Low physical workload	3610 (85)	648 (86)	2962 (85)	0.879
Regular working hours (no shift work)	2303 (76)	447 (84)	1856 (75)	< .0001
Low job strain	1005 (33)	248 (46)	757 (31)	< .0001
High work time control	1048 (32)	280 (46)	768 (29)	< .0001
Good working capacity	2889 (68)	494 (66)	2395 (69)	0.129
Good self-rated health	3220 (76)	566 (75)	2654 (76)	0.430
No psychological distress	3685 (87)	671 (89)	3014 (87)	0.128
No pain	736 (17)	165 (22)	571 (16)	0.0004
No chronic diseases	784 (19)	162 (22)	622 (18)	0.022
Sleep duration over 6.5 h	3041 (72)	488 (65)	2553 (73)	< .0001
No sleep difficulties	3058 (72)	586 (78)	2472 (71)	0.0002
Non-smoker	3783 (91)	666 (89)	3117 (91)	0.048
No risk-use of alcohol	3879 (91)	668 (89)	3211 (92)	0.004
Recommended physical activity	2614 (62)	468 (62)	2146 (62)	0.872
Normal weight	1624 (39)	230 (31)	1394 (41)	< .0001
Extended employment: yes	1233 (29)	263 (35)	970 (28)	0.0002

Unadjusted associations of potential sociodemographic, work- and health-related explanatory factors with extended employment are shown separately for men and women in Online Resource 1. In men and women, participants with no spouse or with a spouse working full-time, those with a non-manual occupation, low physical workload, good working capacity, no pain, no sleep difficulties, and those who were not on part-time retirement were more likely to work beyond their pensionable date. In addition, among women, those with low job strain, high work time control, good self-rated health, risk-use of alcohol, and normal weight were more likely to extend their employment, whereas, among men, those with no chronic diseases were more likely to extend their employment.

To identify potential explanatory factors for the mediation analysis, associations between sex and each outcome are presented in Online Resource 2. Those variables that were not associated with both sex and extended employment (marked with -) were unlikely explanatory factors and, thus, excluded from the mediation analyses. Factors fulfilling the requirements for being an explanatory factor (marked with +) were included in the mediation analyses (i.e. marital status, spouse working full-time, occupational status, part-time retirement, job strain, work time control, pain, chronic diseases, sleep difficulties, alcohol use, and body mass index).

Table 2 shows the results for serial adjustment for potential mediators and their PERM explaining sex differences in extended employment using the traditional mediation analysis. The unadjusted probability for extending employment by more than 6 months beyond the individual pensionable date was 1.29 (95% CI 1.11 – 1.49) among men compared with women. All explanatory factors except for marital status, chronic diseases, and body mass index lead to positive PERM estimates and the largest reductions in RRs were found after adjusting for spouse working full-time (39%), job strain (18%), and work time control (36%). When all mediators (i.e. factors that lead to positive PERM estimates) were accounted for simultaneously, the excess likelihood of men to extend their employment beyond the pensionable date was reduced by 83%.

**Table 2:** Association between sex and extended employment with serial adjustments for potential mediators using traditional mediation analysis (interaction not taken into account) and counterfactual mediation analysis (interaction allowed) (n = 2513)

Adjustments	Traditional mediation analysis		Counterfactual mediation analysis				*p* for interaction^b^
	Men vs. women	PERM	NDE	NIE	Total effect	Proportion mediated	
	RR (95% CI)	%	RR (95% CI)	RR (95% CI)	RR (95% CI)	%	
Unadjusted	1.29 (1.11–1.49)	–	–	–	–	–	–
Pension scheme adjusted	1.29 (1.11–1.49)	Reference	–	–	–	–	–
Married or cohabiting	1.33 (1.15–1.54)	–15.9	1.30 (1.11–1.51)	0.99 (0.94–1.04)	1.29 (1.11–1.49)	–4.3	0.175
Spouse working full-time	1.18 (1.01–1.37)	38.7	1.21 (1.02–1.43)	1.06 (0.98–1.15)	1.29 (1.11–1.49)	27.0	0.461
Non-manual occupation	1.26 (1.09–1.46)	9.8	1.27 (1.10–1.48)	1.01 (0.99–1.03)	1.29 (1.11–1.49)	4.3	0.358
Part-time retirement	1.24 (1.08–1.44)	14.8	1.23 (1.07–1.43)	1.04 (1.01–1.08)	1.29 (1.11–1.49)	18.0	0.518
Low job strain	1.23 (1.06–1.43)	18.2	1.26 (1.08–1.47)	1.02 (0.98–1.06)	1.29 (1.11–1.49)	9.3	0.337
High work time control	1.18 (1.02–1.37)	35.9	1.25 (1.07–1.46)	1.03 (0.98–1.08)	1.29 (1.11–1.49)	12.1	0.050
No pain	1.26 (1.09–1.45)	10.6	1.25 (1.08–1.45)	1.03 (1.00–1.06)	1.29 (1.11–1.49)	13.0	0.729
No chronic diseases	1.29 (1.09–1.49)	–0.4	1.27 (1.10–1.47)	1.01 (0.99–1.03)	1.29 (1.11–1.49)	5.5	0.038
No sleep difficulties	1.27 (1.10–1.47)	4.9	1.27 (1.09–1.47)	1.01 (0.99–1.03)	1.29 (1.11–1.49)	6.3	0.770
No risk-use of alcohol	1.28 (1.11–1.49)	1.2	1.27 (1.10–1.47)	1.01 (0.99–1.03)	1.29 (1.11–1.49)	5.2	0.271
Normal weight (< 25)	1.29 (1.12–1.50)	–2.7	1.30 (1.12–1.50)	0.99 (0.97–1.02)	1.29 (1.11–1.49)	–3.2	0.927
All mediators^a^	1.05 (0.90–1.22)	83.1	–	–	–	–	–

RR = risk ratio, CI = confidence interval, PERM = percentage of excess risk mediated, NDE = natural direct effect, NIE = natural indirect effect

^a^Only factors that were found to be mediators were included in this estimate (i.e. factors mediating excess risk)

^b^*p*-value for sex-mediator interaction

Table 2 also presents the results for counterfactual mediation analysis that allows for interaction between the exposure (sex) and each mediator. The natural direct effect was significant in all models, RRs ranging from 1.21 to 1.30. Only two of the studied 12 potential explanatory factors showed a statistically significant natural indirect effect. Of the association between sex and extended employment, 18% was explained by the lower likelihood of men to be on part-time retirement before the pensionable date than women (NIE 1.04, 95% CI 1.01–1.08). Similarly, men’s lower likelihood to perceive pain symptoms compared with women explained 13% of the association (NIE 1.03, 95% CI 1.00–1.06).

We found a borderline significant sex-mediator interaction (*p* = 0.050) between sex and work time control and a statistically significant interaction between sex and chronic diseases (*p* = 0.038) in the counterfactual models predicting extended employment. The association of high work time control with extended employment was stronger for women (RR 1.54, 95% CI 1.34–1.79) than men (RR 1.15, 95% CI 0.89–1.49), whereas the association of having no chronic diseases was stronger for men (RR 1.52, 95% CI 1.17–1.98) than women (RR 1.08, 95% CI 0.90–1.30) when adjusted for pension scheme. As a result of allowing the interactions, the proportion mediated was lower for work time control (12% vs. 36%) and higher for chronic diseases (6% vs. -0.4%) when compared to the traditional mediation analysis. Allowing interaction between exposure and mediator resulted also in the proportion mediated being lower for spouse working full-time (27% vs. 39%) and job strain (9% vs. 18%), and higher for part-time retirement (18% vs. 14%) in the counterfactual vs. traditional mediation analysis.

To examine the robustness of the associations observed, we repeated the main analyses using an alternative cut-off point of over 12 months beyond the pensionable date to define extension of employment. Factors fulfilling the requirements for being an explanatory factor for this longer extension of employment were largely similar to those in the main analyses (data not shown). Job strain and sleep difficulties were dropped out from the mediation analyses, as they were not associated with the alternative extension of employment variable and sleep duration was included as a potential explanatory factor as it was associated with this variable among women. Findings for the extension of employment of over 12 months largely replicated those in the main analyses (see Online Resource 3). Except for marital status and body mass index, all the explanatory factors lead to positive PERM estimates, and the excess likelihood of men to extend their employment for over 12 months beyond the pensionable date was reduced by 86% when all potential mediators were accounted for simultaneously. There were no significant sex differences in mediators according to counterfactual models predicting the extension of employment of over 12 months.

## Discussion

This prospective study of Finnish public sector employees examined differences between men and women in working beyond the pensionable age. Men were 1.29-times more likely to extend their employment by over 6 months than women. As much as 83% of the excess likelihood of men to extend employment was explained by sex differences in socio-demographic, work- and health-related mediators, such as working status of a spouse, part-time retirement, job strain, work time control, and pain symptoms. According to traditional mediation analyses, each of these factors explained 11% to 39% of the association. The robustness of these findings was supported by highly similar findings in supplementary analyses with an alternative definition of the extension of employment based on a 12-month cut-off.

According to the traditional mediation analysis, the main mediators for sex differences in extended employment were working status of a spouse (mediated 39% of the excess risk) and work time control (mediated 36% of the excess risk). Previous studies have reported associations between the spouse’s opinion towards continued working and the likelihood of working beyond retirement (de Wind et al. 2018; Scharn et al. 2017) and some studies have found that the working status of a spouse predicts extended employment (Gonzales and Nowell 2017). In our study, men were much more likely to have a spouse working full-time than women (46% vs. 17%), which might reflect age differences within couples with women being generally younger. Among both men and women, extending employment beyond the pensionable age was more likely among those with a spouse working full-time than among those with a spouse not working full-time or those without a spouse. High work time control, along with other indicators of more favorable psychosocial work environment, has been associated with extended employment in previous studies (Virtanen et al. 2014, 2017). In our study, men were more likely to have high work time control than women (46% vs. 29%) and, thus, our findings are consistent with previous research.

We complemented traditional analyses by counterfactual mediation analysis, which decomposes the mediation effect into direct and indirect effects. All the examined explanatory factors had a natural direct effect on extended employment of over 6 months beyond the pensionable date. This means that in a scenario in which the mediator is at the same level among men and women, men are, depending on the explanatory factor, from 1.21 to 1.30 times more likely to extend their employment compared with women. However, a natural indirect effect was observed only for part-time retirement and pain. This indicates that men have a greater likelihood of extended employment solely because of the greater likelihood for not being on part-time retirement or not having pain symptoms. Although part-time retirement is provided for employees to encourage them to remain in the working life (Hermansen 2015), shifting from full-time to part-time work has instead shortened working lives in European countries (Hess et al. 2018). Women may choose part-time retirement, for example, because they are more often engaged in caregiving of their parents or taking care of their grandchildren than men. Furthermore, part-time retirement could be chosen due to health reasons, for example, due to not having the health capacity needed for working full-time. Another factor that may limit working capacity is pain. To the best of our knowledge, this is the first study to show that employees without pain symptoms had increased likelihood of extended employment beyond the pensionable age. Pain, both acute and chronic, and especially when associated with a long-standing illness, is a major factor contributing to disability retirement (Saastamoinen et al. 2012); and therefore, pain symptoms may restrict extending employment. Our findings warrant further research on whether the greater likelihood of men to extend employment beyond the pensionable age is attributable to poorer health among women in the public sector in Finland. Future intervention studies are also needed in order to examine whether targeting these factors could improve working life participation among aging female workers and thus diminish the sex differences in the likelihood of extending employment.

Unlike the traditional mediation method, the counterfactual analysis takes into account the possible interaction between the exposure (here sex) and mediators (here explanatory factors) (Valeri and VanderWeele 2013). Examining the potential impact of the interaction is important, as factors such as socioeconomic status (especially public sector employment) (Riekhoff and Järnefelt 2017), poor mental health (Demou et al. 2017), and high physical work demands (Andersen et al. 2020) have been suggested to have a more marked role in the timing of retirement among women than among men. Different factors may indeed motivate men and women to continue working beyond the pensionable age. We observed a statistically significant interaction only between sex and work time control and sex and chronic diseases. High work time control predicted greater likelihood for extended employment when compared to those with low work time control only among women. Having no chronic diseases, on the other hand, predicted greater likelihood for extended employment only among men when compared to those with chronic diseases. No other statistically significant interactions were observed between sex and explanatory factors in predicting over 6- or 12-month extension of employment. It is possible that this study, with no more than 2,500 participants included in the mediation analyses, had limited powered to detect interactions (Andersen et al. 2020). Nonetheless, these findings highlight that some factors may have a dissimilar role in postponing retirement for men and women. Thus, using methods that allow the exposure-mediator interaction, such as the counterfactual mediation analysis, is crucial when examining the contribution of different explanatory factors for sex differences in extended employment.

Some factors that have been associated with extended employment in previous studies, such as perceived working ability (Virtanen et al. 2017), physical workload (Andersen et al. 2020; Virtanen et al. 2017), and self-rated health (Demou et al. 2017) were not included as explanatory factors in this study, as they were not associated with sex and therefore could not have contributed to the sex differences in extended employment. In addition, some of the factors that have previously been associated with the timing of retirement, such as caregiving (Stoiko and Strough 2019) and mental health (assessed with the measure of psychological distress in this study) (Demou et al. 2017; Virtanen et al. 2014), were not associated with sex or extended employment in this study and were thus excluded from the mediation analyses. The absence of these association was unexpected, as women have been considered to be engaged in caregiving more than men, and this has been suggested as one of the reasons for sex differences in the timing of retirement (Stoiko and Strough 2019). In our study, sex differences and the number of participants reporting that they provided care for a family member were small (13% among men and 16% among women), which might have limited statistical power to observe associations with extended employment. Furthermore, we did not have information on the intensity or type of caregiving provided, which may also play a role in the association between caregiving and labor market participation (Heitmueller 2007). Mental health has been suggested to be more likely associated with the timing of retirement among women than among men (Demou et al. 2017), but the small number of those who experienced psychological distress in our study (11% among men and 13% among women) limited statistical power in our study to detect such associations.

The main strengths of this study include the prospective design and the comparison of two different methods for quantifying mediation and examining the contribution of explanatory factors for sex differences in extended employment. Due to the prospective design of the study, we only examined work conditions of the employees *before* the pensionable age, and thus we did not take into account the work characteristics the employees actually had when working *after* the pensionable age, which has been pointed out previously as a limiting factor (Wahrendorf et al. 2017). In this study, the work conditions could be thought to be quite similar at baseline (i.e. before the pensionable date) and after the pensionable age, as this study concerns *continued* employment rather than the so-called bridge employment, which is often used to refer to employment one engages in after retiring from their career job (Beehr and Bennett 2015). Furthermore, work conditions, such as job strain and work time control have been observed to remain relatively stable over time (Åhlin et al. 2018).

Our study sample represents employees of the municipal sector in Finland, and thus, the findings of this study may not be generalizable to other working sectors or to countries with different pension systems. In addition, the sex distribution of our study (82% women vs. 18% men), although corresponding to the sex distribution of the Finnish public sector, was unbalanced, and the small number of men may have limited the possibilities to detect all potential sex differences in the study. Further research with larger sample sizes and more balanced study population in terms of sex distribution is needed to corroborate our findings. Furthermore, due to the observational nature of our study, it was not possible to eliminate residual confounding. Thus, if there are unmeasured or deficiently measured confounders, the direct and indirect effect estimates are biased and do not have a casual interpretation (Valeri and VanderWeele 2013). The sociodemographic, work- and health-related factors considered in this study explained little over 80% of the association between sex and working beyond pensionable age, and thus, further research is needed to determine the unexplained proportion of this association.

In conclusion, among public sector employees in Finland, men were more likely than women to extend their employment beyond the pensionable age by more than six months. The greater likelihood of men to extended employment was contributed the most by men having more often a full-time working spouse, low job strain, high work time control, reporting no pain, and being less often on part-time retirement before the pensionable age than women. Thus, along with sex differences in sociodemographic and health-related factors, our results indicate potential inequalities between men and women in working conditions. In the public sector in Finland, women are more likely to work in occupations with less favorable psychosocial working conditions. In order to encourage women’s participation in working life after the pensionable age and to reduce sex differences, policies improving psychosocial working conditions among women may need to be established. Furthermore, policies aiming at encouraging working life participation after the pensionable age should consider that different factors may motivate men and women to continue working at older ages. This study suggests high work time control is a stronger predictor of extended employment in women than men whereas having no chronic diseases is a stronger predictor of extended employment in men than women.

## Supplementary Information

Below is the link to the electronic supplementary material.

Supplementary file1 (DOCX 24 KB)

Supplementary file2 (DOCX 22 KB)

Supplementary file3 (DOCX 24 KB)

## Data Availability

Anonymized partial datasets of the Finnish Retirement and Aging Study are available by application with bona fide researchers with an established scientific record and bona fide organizations. For more information, please contact prof. Sari Stenholm, sari.stenholm[at]utu.fi.

## Code availability

The SAS codes used in this study are available by application with bona fide researchers with an established scientific record and bona fide organizations. For more information, please contact Dr. Saana Myllyntausta, saana.myllyntausta[at]utu.fi.
