# Supplementary material for: Why do men extend their employment beyond pensionable age more often than women? a cohort study
Source: Eur J Ageing. 2021 Dec 5;19(3):599–608. doi: 10.1007/s10433-021-00663-1 (PMC9424425; doi:10.1007/s10433-021-00663-1)
Supplement: Supplementary file 1 — Supplementary file1 (DOCX 24 KB) [file 10433_2021_663_MOESM1_ESM.docx]

# ONLINE RESOURCES

**European Journal of Ageing

Why do men extend their employment beyond pensionable age more often than women? A cohort study**

Saana Myllyntausta^1,2,3^ (Orcid ID: 0000-0002-6503-3829), Marianna Virtanen ^4,5^ (0000-0001-8361-3301), Jaana Pentti^2,3,6^, Mika Kivimäki^6,7,8^ (0000-0002-4699-5627), Jussi Vahtera^2,3^ (0000-0002-6036-061X), Sari Stenholm^2,3^ (0000-0001-7560-0930)

*^1^ Department of Psychology and Speech-Language Pathology, University of Turku, Turku, Finland
^2^ Department of Public Health, University of Turku and Turku University Hospital, Turku, Finland*

*^3^ Centre for Population Health Research, University of Turku and Turku University Hospital, Turku, Finland
^4^ School of Educational Sciences and Psychology, Psychology, University of Eastern Finland, Joensuu, Finland*

*^5^Division of Insurance Medicine, Karolinska Institutet, Stockholm, Sweden*

*^6^ Clinicum, Faculty of Medicine, University of Helsinki, Helsinki, Finland
^7^ Department of Epidemiology and Public Health, University College London Medical School, London, United Kingdom ^8^ Finnish Institute of Occupational Health, Helsinki, Finland*

**Corresponding author:**
Dr. Saana Myllyntausta
Department of Psychology and Speech-Language Pathology, University of Turku
E-mail: [saana.myllyntausta@utu.fi](mailto:saana.myllyntausta@utu.fi)

**Online Resource 1** – Predictors of extended employment by sex.

|  | **Men** | | | | **Women** | | | |
| --- | --- | --- | --- | --- | --- | --- | --- | --- |
| **Characteristics** | **% with extended employment** | **RR^a^** | **95% CI** | | **% with extended employment** | **RR^a^** | **95% CI** | |
| Married or cohabiting: yes vs. no | 34/35 | 0.95 | 0.74 | 1.24 | 25/33 | 0.75 | 0.67 | 0.84 |
| Spouse working full-time: yes vs. no | 39/31 | 1.27 | 1.05 | 1.55 | 34/26 | 1.31 | 1.15 | 1.49 |
| Not providing care vs. providing care | 35/30 | 1.18 | 0.85 | 1.62 | 28/27 | 1.02 | 0.88 | 1.19 |
| Non-manual occupation vs. service or manual occupation | 38/26 | 1.46 | 1.15 | 1.85 | 31/21 | 1.48 | 1.31 | 1.68 |
| Part-time retirement: yes vs. no | 15/38 | 0.40 | 0.26 | 0.62 | 13/32 | 0.43 | 0.35 | 0.52 |
| Low physical workload: yes vs. no | 37/22 | 1.67 | 1.16 | 2.41 | 29/22 | 1.34 | 1.12 | 1.59 |
| Regular working hours (no shift work) vs. shift work | 34/30 | 1.14 | 0.80 | 1.63 | 27/25 | 1.08 | 0.92 | 1.26 |
| Low job strain: yes vs. no | 37/32 | 1.15 | 0.91 | 1.46 | 32/24 | 1.32 | 1.16 | 1.51 |
| High work time control: yes vs. no | 38/31 | 1.23 | 0.99 | 1.53 | 36/24 | 1.47 | 1.30 | 1.67 |
| Good working capacity: yes vs. no | 39/25 | 1.60 | 1.26 | 2.03 | 32/17 | 1.88 | 1.63 | 2.17 |
| Good self-rated health: yes vs. no | 36/29 | 1.27 | 0.99 | 1.63 | 30/20 | 1.53 | 1.32 | 1.77 |
| No psychological distress vs. psychological distress | 35/34 | 1.03 | 0.75 | 1.40 | 28/27 | 1.05 | 0.89 | 1.23 |
| No pain vs. (mild or severe) pain | 44/32 | 1.36 | 1.10 | 1.67 | 34/26 | 1.29 | 1.13 | 1.46 |
| No chronic diseases vs. chronic diseases | 45/32 | 1.41 | 1.15 | 1.74 | 29/28 | 1.04 | 0.91 | 1.19 |
| Sleep duration over 6.5 hours vs. less | 33/38 | 0.86 | 0.70 | 1.04 | 27/29 | 0.92 | 0.81 | 1.03 |
| No sleep difficulties vs. sleep difficulties | 37/28 | 1.32 | 1.01 | 1.72 | 29/25 | 1.14 | 1.00 | 1.28 |
| Non-smoker: yes vs. no | 35/35 | 0.99 | 0.73 | 1.34 | 27/30 | 0.93 | 0.78 | 1.12 |
| No risk-use of alcohol vs. risk-use of alcohol | 34/42 | 0.81 | 0.62 | 1.07 | 27/33 | 0.83 | 0.69 | 0.99 |
| Recommended physical activity (≥14 MET): yes vs. no | 34/36 | 0.95 | 0.78 | 1.16 | 29/26 | 1.08 | 0.97 | 1.21 |
| Normal weight (<25): yes vs. no | 38/33 | 1.14 | 0.93 | 1.40 | 30/26 | 1.12 | 1.01 | 1.25 |

^a^ Unadjusted. RR = risk ratio, CI = confidence interval
