# Supplementary material for: Why do men extend their employment beyond pensionable age more often than women? a cohort study
Source: Eur J Ageing. 2021 Dec 5;19(3):599–608. doi: 10.1007/s10433-021-00663-1 (PMC9424425; doi:10.1007/s10433-021-00663-1)
Supplement: Supplementary file 2 — Supplementary file2 (DOCX 22 KB) [file 10433_2021_663_MOESM2_ESM.docx]

# ONLINE RESOURCES

**European Journal of Ageing

Why do men extend their employment beyond pensionable age more often than women? A cohort study**

Saana Myllyntausta^1,2,3^ (Orcid ID: 0000-0002-6503-3829), Marianna Virtanen ^4,5^ (0000-0001-8361-3301), Jaana Pentti^2,3,6^, Mika Kivimäki^6,7,8^ (0000-0002-4699-5627), Jussi Vahtera^2,3^ (0000-0002-6036-061X), Sari Stenholm^2,3^ (0000-0001-7560-0930)

*^1^ Department of Psychology and Speech-Language Pathology, University of Turku, Turku, Finland
^2^ Department of Public Health, University of Turku and Turku University Hospital, Turku, Finland*

*^3^ Centre for Population Health Research, University of Turku and Turku University Hospital, Turku, Finland
^4^ School of Educational Sciences and Psychology, Psychology, University of Eastern Finland, Joensuu, Finland*

*^5^Division of Insurance Medicine, Karolinska Institutet, Stockholm, Sweden*

*^6^ Clinicum, Faculty of Medicine, University of Helsinki, Helsinki, Finland
^7^ Department of Epidemiology and Public Health, University College London Medical School, London, United Kingdom ^8^ Finnish Institute of Occupational Health, Helsinki, Finland*

**Corresponding author:**
Dr. Saana Myllyntausta
Department of Psychology and Speech-Language Pathology, University of Turku
E-mail: [saana.myllyntausta@utu.fi](mailto:saana.myllyntausta@utu.fi)

**Online Resource 2 –** Association of potential explanatory factors with sex and outcome (extended employment).

|  | **With sex** | **With outcome** | **Status as explanatory factor** |
| --- | --- | --- | --- |
| Married or cohabiting | + | + opposite | + |
| Spouse working full-time | + | + | + |
| Not providing care for a family member | - | - | - |
| Non-manual occupation | + | + | + |
| Part-time retirement | + | + opposite | + |
| Low physical workload | - | + | - |
| Regular working hours (no shift work) | + | - | - |
| Low job strain | + | + | + |
| High work time control | + | + | + |
| Good working capacity | - | + | - |
| Good self-rated health | - | + | - |
| No psychological distress | - | - | - |
| No pain | + | + | + |
| No chronic diseases | + | + | + |
| Sleep duration over 6.5 hours | + | - | - |
| No sleep difficulties | + | + | + |
| Non-smoker | + | - | - |
| No risk-use of alcohol | + | + opposite | + |
| Recommended physical activity (≥14 MET) | - | - | - |
| Normal weight (<25) | + | + | + |

- Does not fulfill requirements for being an explanatory factor
+ Fulfills requirements for being an explanatory factor
