# Supplementary material for: Why do men extend their employment beyond pensionable age more often than women? a cohort study
Source: Eur J Ageing. 2021 Dec 5;19(3):599–608. doi: 10.1007/s10433-021-00663-1 (PMC9424425; doi:10.1007/s10433-021-00663-1)
Supplement: Supplementary file 3 — Supplementary file3 (DOCX 24 KB) [file 10433_2021_663_MOESM3_ESM.docx]

# ONLINE RESOURCES

**European Journal of Ageing

Why do men extend their employment beyond pensionable age more often than women? A cohort study**

Saana Myllyntausta^1,2,3^ (Orcid ID: 0000-0002-6503-3829), Marianna Virtanen ^4,5^ (0000-0001-8361-3301), Jaana Pentti^2,3,6^, Mika Kivimäki^6,7,8^ (0000-0002-4699-5627), Jussi Vahtera^2,3^ (0000-0002-6036-061X), Sari Stenholm^2,3^ (0000-0001-7560-0930)

*^1^ Department of Psychology and Speech-Language Pathology, University of Turku, Turku, Finland
^2^ Department of Public Health, University of Turku and Turku University Hospital, Turku, Finland*

*^3^ Centre for Population Health Research, University of Turku and Turku University Hospital, Turku, Finland
^4^ School of Educational Sciences and Psychology, Psychology, University of Eastern Finland, Joensuu, Finland*

*^5^Division of Insurance Medicine, Karolinska Institutet, Stockholm, Sweden*

*^6^ Clinicum, Faculty of Medicine, University of Helsinki, Helsinki, Finland
^7^ Department of Epidemiology and Public Health, University College London Medical School, London, United Kingdom ^8^ Finnish Institute of Occupational Health, Helsinki, Finland*

**Corresponding author:**
Dr. Saana Myllyntausta
Department of Psychology and Speech-Language Pathology, University of Turku
E-mail: [saana.myllyntausta@utu.fi](mailto:saana.myllyntausta@utu.fi)

**Online Resource 3 –** Association between sex and extension of employment of over 12 months with serial adjustments for potential mediators using traditional mediation analysis (interaction not taken into account) and counterfactual mediation analysis (interaction allowed) (n = 2,819).

|  | **Traditional  mediation analysis** | |  | **Counterfactual mediation analysis** | | | | |
| --- | --- | --- | --- | --- | --- | --- | --- | --- |
|  | **Men vs. women** | **PERM** |  | **NDE** | **NIE** | **Total effect** | **Proportion mediated** |  |
| **Adjustments** | **RR (95% CI)** | **%** |  | **RR (95% CI)** | **RR (95% CI)** | **RR (95% CI)** | **%** | **p for interaction^b^** |
| Unadjusted | 1.27 (1.04–1.56) | - |  | - | - | - | - | - |
|  | 1.24 (1.02–1.52) | reference |  | - | - | - | - | - |
| Married or cohabiting | 1.30 (1.06–1.59) | -24.1 |  | 1.22 (1.01–1.55) | 0.99 (0.93–1.06) | 1.25 (1.02–1.52) | -3.4 | 0.204 |
| Spouse working full-time | 1.14 (0.93–1.41) | 41.7 |  | 1.12 (0.89–1.41) | 1.12 (0.997–1.25) | 1.25 (1.03–1.53) | 51.4 | 0.732 |
| Non-manual occupation | 1.20 (0.99–1.47) | 16.7 |  | 1.21 (0.99–1.48) | 1.02 (0.99–1.05) | 1.24 (1.01–1.51) | 11.9 | 0.828 |
| Part-time retirement | 1.20 (0.98–1.46) | 17.9 |  | 1.20 (0.99–1.47) | 1.03 (1.00–1.07) | 1.24 (1.02–1.52) | 16.8 | 0.848 |
| High work time control | 1.17 (0.96–1.43) | 29.8 |  | 1.15 (0.93–1.42) | 1.09 (1.02–1.18) | 1.26 (1.03–1.53) | 42.4 | 0.557 |
| No pain | 1.23 (1.00–1.50) | 6.8 |  | 1.20 (0.98–1.48) | 1.02 (0.995–1.06) | 1.23 (1.01–1.51) | 12.8 | 0.322 |
| No chronic diseases | 1.24 (1.02–1.52) | 0.5 |  | 1.23 (1.01–1.51) | 1.01 (0.99–1.03) | 1.24 (1.02–1.52) | 4.0 | 0.228 |
| Sleep duration over 6.5 hours | 1.22 (1.00–1.50) | 7.9 |  | 1.23 (1.00–1.50) | 1.01 (0.98–1.04) | 1.24 (1.02–1.52) | 5.6 | 0.773 |
| No risk-use of alcohol | 1.23 (1.01–1.50) | 4.8 |  | 1.21 (0.99–1.49) | 1.03 (0.99–1.06) | 1.24 (1.02–1.52) | 12.5 | 0.303 |
| Normal weight (<25) | 1.27 (1.04–1.55) | -12.0 |  | 1.26 (1.03–1.55) | 0.99 (0.95–1.02) | 1.24 (1.02–1.52) | -7.8 | 0.534 |
| All mediators^a^ | 1.03 (0.84–1.27) | 86.4 |  | - | - | - | - | - |

RR = risk ratio, CI = confidence interval, PERM = percentage of excess risk mediated, NDE = natural direct effect, NIE = natural indirect effect ^a^ Only factors that were found to be mediators were included in this estimate (i.e. factors mediating excess risk).
^b^ p-value for sex-mediator interaction
